# BMAL1 knockdown triggers different colon carcinoma cell fates by altering the delicate equilibrium between AKT/mTOR and P53/P21 pathways

**DOI:** 10.18632/aging.103124

**Published:** 2020-05-10

**Authors:** Yuan Zhang, Aurore Devocelle, Lucas Souza, Adlen Foudi, Sabrina Tenreira Bento, Christophe Desterke, Rachel Sherrard, Annabelle Ballesta, Rene Adam, Julien Giron-Michel, Yunhua Chang

**Affiliations:** 1 INSERM, UMR935, Malignant and Therapeutic Stem Cells Models, Villejuif, France; 2 Paris-Saclay University, Saint-Aubin, France; 3 INSERM, UMR1197 Interactions between Stem Cells and Their Niches in Physiology, Tumors and Tissue Repair, Villejuif, France; 4 Hôpital Paul Brousse AP-HP, Villejuif, France; 5 Sorbonne Université and CNRS, Institut de Biologie Paris Seine, UMR8256 Biological Adaptation and Aging (B2A), Paris, France

**Keywords:** BMAL1, mTOR, P53, senescence, colorectal cancer1

## Abstract

Dysregulation of the circadian timing system (CTS) frequently appears during colorectal cancer (CRC) progression. In order to better understand the role of the circadian clock in CRC progression, this study evaluated *in vitro* how knockdown of a core circadian protein BMAL1 (BMAL1-KD) influenced the behavior of two primary human CRC cell lines (HCT116 and SW480) and a metastatic CRC cell line (SW620).

Unexpectedly, BMAL1-KD induced CRC cell-type specific responses rather than the same phenomenon throughout. First, BMAL1-KD increased AKT/mTOR activation in each CRC cell line, but to different extents. Second, BMAL1-KD-induced P53 activation varied with cell context. In a wild type P53 background, HCT116 BMAL1-KD cells quickly underwent apoptosis after shBMAL1 lentivirus transduction, while surviving cells showed less P53 but increased AKT/mTOR activation, which ultimately caused higher proliferation. In the presence of a partially functional mutant P53, SW480 BMAL1-KD cells showed moderate P53 and mTOR activation simultaneously with cell senescence. With a moderate increased AKT but unchanged mutant P53 activation, SW620 BMAL1-KD cells grew faster.

Thus, under different CRC cellular pathological contexts, BMAL1 knockdown induced relatively equal effects on AKT/mTOR activation but different effects on P53 activation, which finally triggered different CRC cell fates.

## INTRODUCTION

The circadian timing system (CTS) exists in most living organisms with a basic molecular frame preserved from fungi to Drosophila and humans. This system coordinates behavior of the whole organism, including physiology and metabolism, with environmental cycles of 24h. In mammals, the suprachiasmatic nuclei of the hypothalamus coordinate circadian rhythms via peripheral molecular clocks composed of at least fifteen genes that are expressed in every cell. Expression of these clock gene is regulated by transcription factors organized in positive (BMAL1 and CLOCK) or negative (PER and CRY) feedback loops. Briefly, the transcriptional activator complex of BMAL1/CLOCK activates transcription of its target genes inducing expression of CRY and PER proteins, which in turn repress their own transcription through their interactions with the BMAL1/CLOCK heterodimer. The BMAL1/CLOCK heterodimer also activates the expression of REV-ERB α/β (NR1D1 and NR1D2) and ROR α/β/γ, which repress and activate *BMAL1* transcription, respectively [1, 2]. Thus *BMAL1* is central to circadian timing and is the only clock gene whose deletion causes an immediate loss of behavioral circadian rhythmicity [1, 3].

This molecular circadian clock regulates multiple cellular processes, with ~43% of mammalian protein-coding genes showing rhythmic expression at least in one organ [4]. Also, 25% of protein phosphorylation [5] and nuclear accumulation of over 10% of nuclear proteins [6] exhibit circadian oscillation. Thus, by regulating many fundamental cellular processes, such as cell cycle, metabolism, senescence, apoptosis and DNA damage response, an intact circadian clock plays a crucial role in maintaining normal cell life and its dysfunction perturbs numerous cellular activities, thereby becoming a risk factor for disease, such as cancer [7, 8].

The link between circadian rhythms and cancer is indicated by an increased risk of cancer in people whose daily rhythms are disturbed by shift work or insufficient sleep [9]. Furthermore, circadian rhythmicity is often dysregulated in cancer patients and associated with poor prognosis and early mortality [10–13]. Although the BMAL1 exhibits a globally repressive function in many tumors, some studies also reveal that BMAL1 might favor tumorigenesis under certain circumstances. For example, compared to healthy tissue, colorectal cancers (CRC) often display higher CLOCK or BMAL1 expression, which is associated with liver metastasis and poorly differentiated or late-stage CRC cancer [14–16]. In addition, the majority of malignant pleural mesothelioma (MPM) cell lines, and a subset of MPM clinical specimens, expressed more BMAL1 compared to their non-cancer controls (non-tumorigenic mesothelial cell line - MeT-5A - and normal parietal pleura, respectively). Moreover, BMAL1 knockdown (BMAL1-KD) in MPM cell lines reduced cell growth and induced apoptosis [17, 18]. Therefore, the relationship between BMAL1 and cancer development is complex and requires deeper investigation to reveal molecular mechanistic insights.

CRC is one of the most common cancers. In 2012, there were 1.4 million new cases and693,900 deaths worldwide from the disease [19]. In this study, we investigated the influence of BMAL1 deficiency in CRC cell behavior in order to better understand the role of the circadian clock in colon cancer development at cellular and molecular levels. We have selected two primary colorectal adenocarcinoma cell lines, HCT116 and SW480, and a metastatic CRC cell line derived from the same patient as SW480 cells (SW620). Both primary CRC cell lines, HCT116 and SW480, express core-clock genes with circadian oscillation, whereas this oscillation is severely diminished in the metastatic cell line SW620 [20, 21, 22]. Using these three cell lines, we knocked down *BMAL1* expression by shRNA to investigate the influence of BMAL1 deficiency on CRC cell behavior.

Our results revealed that BMAL1-KD activated AKT/mTOR similarly in the three CRC cell lines (HCT116, SW480 or SW620), but had different effects on P53 activation. mTOR signaling is an evolutionarily conserved nutrient sensing pathway and a central regulator of mammalian metabolism. It has been hypothesized that increased mTOR activity could direct cell fate towards quiescence, cell death or senescence under varying P53 activation and P21 expression status [23–26]. Here, by altering the delicate equilibrium between AKT/mTOR and P53/P21 pathways, BMAL1-KD modulates CRC cell fates on the basis of their distinct cellular context.

## RESULTS

### Decreased BMAL1 altered expression of some circadian genes in primary CRC cell lines

Three CRC cell lines, two primary cell lines (HCT116 and SW480) and a metastatic cell line SW620, were transduced with lentiviruses encoding a scrambled shRNA (shScr) or a shRNA targeting BMAL1 (shBMAL1). After transduction, cells were selected by one-week puromycin treatment to remove non-transduced cells. Successful transduction was confirmed by flow cytometry of GFP expressing cells. The GFP positive cell population was used immediately for analysis as BMAL1-KD or control (Ctr) cells.

**Figure 1:**
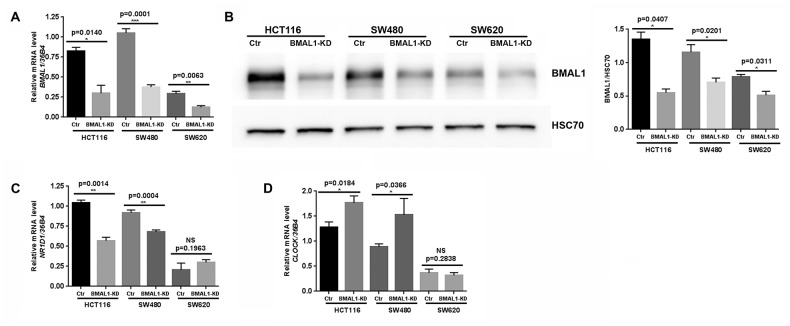
**Lentiviral Sh*BMAL1* decreased BMAL1 expression in three CRC cell lines but only altered expression of some circadian genes in primary CRC cell lines.** (**A**) Effect of sh*BMAL1* on BMAL1 mRNA level was ascertained by quantitative RT-PCR. *36B4* was used as a quantitative reference (n=5; *p<0.05; ***p<0.001; ****p<0.0001). (**B**) Effect of sh*BMAL1* on the level of BMAL1 protein was ascertained by Western-blot. *Left,* a representative immunoblot is shown. *Right,* Bar charts represent BMAL1 expression normalized to HSC70 (n=3; *p<0.05). Data are shown as mean ± SEM. (**C**) Quantitative RT-PCR revealed decreased expression of *NR1D1* in two primary BMAL1-KD CRC cell lines (HCT116 and SW480) but not in the metastatic CRC cell line SW620. (**D**) Quantitative RT-PCR revealed increased expression of *CLOCK* in two primary BMAL1-KD CRC cell lines but not in the metastatic CRC cell line SW620.

BMAL1 expression was significantly decreased compared to control at mRNA (Figure 1A, qRT-PCR) and protein levels (Figure 1B, Western blot) in all three BMAL1-KD cell lines, despite the fact that the two primary CRC cell lines exhibited much higher BMAL1 expression than the metastatic CRC cell line SW620.

Expression of BMAL1 target circadian genes (*NR1D1, PER2, CRY1* and *CRY2*) and its functional partner *CLOCK,* were also analyzed following BMAL1-KD. In the two primary BMAL1-KD CRC cell lines (HCT116 and SW480), but not in BMAL1-KD SW620 cells, *NR1D1* expression was reduced and *CLOCK* expression was increased (Figure 1C, 1D). No significant modification of *PER2*, *CRY1* or *CRY2* expression was identified in any of the three cell lines (Data not shown).

BMAL1-KD thus modified circadian gene expression profile in two primary CRC cell lines (HCT116 and SW480) but not in the metastatic CRC cell line SW620.

### BMAL1-KD increased AKT/mTOR activation in primary CRC cell lines

BMAL1 knockout mice manifest increased mTORC1 (mammalian target of rapamycin complex 1) activity and associated premature aging [27]. We thus investigated whether BMAL1-KD could modify AKT/mTOR signaling in CRC cell lines.

**Figure 2:**
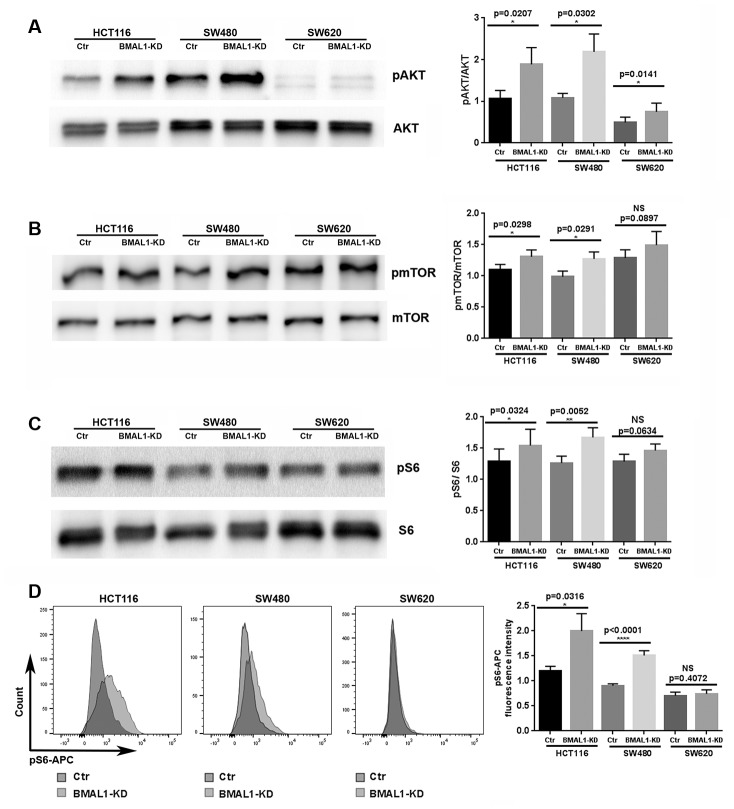
**BMAL1-KD increased AKT/mTOR activation to varying degrees in the CRC cell lines.** (**A**) Western-blot analysis revealed that BMAL1-KD increased AKT phosphorylation in the three CRC cell lines (n=5; *p<0.05; **p<0.01). The ratio of phosphorylated AKT to total AKT was used to indicate AKT activation level. (**B**) Western-blot analysis revealed that BMAL1-KD increased mTOR phosphorylation in HCT116 and SW480 (n=6; *p<0.05) but not in SW620 cells. The ratio between phosphorylated mTOR and total mTOR was used to indicate mTOR activation level. (**C**) Western-blot analysis revealed that knockdown BMAL1 increased 40S Ribosomal protein S6 phosphorylation in HCT116 and SW480 (n=6; ***p<0.001) but not in SW620 cells. The ratio between phosphorylated S6 and total S6 was used to evaluate mTOR activity. (A-C): *Left*, a representative immunoblot of independent experiments. *Right*, Bar charts represent the target protein expression level normalized to protein loading controls. (**D**) Flow cytometry analysis revealed increased phosphorylated S6 in HCT116 BMAL1-KD (*p<0.05) and SW480 BMAL1-KD (****p<0.0001) cells but not in SW620 BMAL1-KD cells compared to their proper controls. *Left,* representative staining of 7 independent experiments is shown. *Right,* Graphs represented the mean fluorescence intensity value of phosphorylated S6-APC (n=7). All data are shown as means ± SEM.

BMAL1-KD increased AKT activation in colon cancer cell lines. Compared to their controls, the percentage of AKT phosphorylation, measured by the ratio between phosphorylated AKT (pAKT) and total AKT, was greatly increased in the primary CRC lines, HCT116 BMAL1-KD (p=0.0207) and SW480 BMAL1-KD (p=0.0302), and slightly increased in the metastatic SW620 BMAL1-KD (p=0.0141) cells (Figure 2A).

Moreover, mTOR activation, measured by the ratio between phosphorylated mTOR (pmTOR) and total mTOR, was significantly increased in HCT116 BMAL1-KD cells (p=0.0298) and in SW480 BMAL1-KD cells (p=0.0291) in comparison to their respective controls. However, SW620 BMAL1-KD cells only showed trend to increased mTOR activation (p=0.0897) (Figure 2B).

To further evaluate mTOR activity, we measured the phosphorylation of 40S Ribosomal protein S6, a major mTOR effector. Western blot analysis revealed a significant increase of S6 phosphorylation (pS6/S6 total) in HCT116 BMAL1-KD cells (p=0.0324) and in SW480 BMAL1-KD cells (p=0.0052) compared to controls. However, only a trend to increased phosphorylation was found in SW620 BMAL1-KD (p=0.0634; Figure 2C). These results were further confirmed by flow cytometry analysis, which revealed that the mean fluorescence intensity of pS6-APC staining increased significantly in HCT116 BMAL1-KD cells (p=0.0316) and in SW480 BMAL1-KD cells (p<0.0001), but not in SW620 BMAL1-KD cells (p=0.4027) compared to their own controls (Figure 2D).

### BMAL1-KD induced different cell proliferation patterns in CRC cell lines

**Figure 3:**
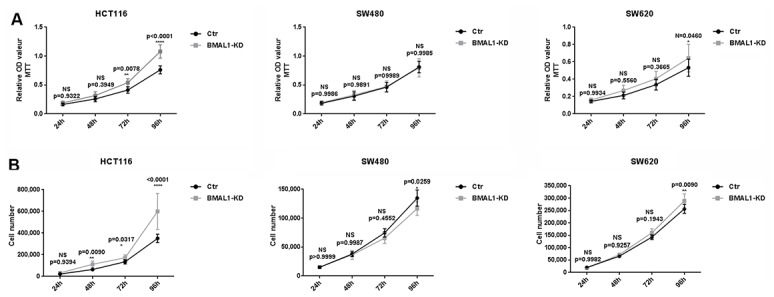
**BMAL1-KD induced different cell proliferation patterns in CRC cell lines.** MTT cell proliferation assay (**A**) and cell counts (**B**) were used to examine BMAL1-KD and control cells’ proliferation rate for 96h. Stable HCT116 BMAL1-KD but not SW480 BMAL1-KD cells exhibited significantly higher cell counts compared to their control. SW620 BMAL1-KD cells only showed faster growth at 96h. (n=8; *p<0.05; **p<0.01; ****p<0.0001). Error bar represented ± SEM.

The activation of mTOR pathway by AKT kinase is implicated in many fundamental cell functions, such as survival, proliferation and growth [28]. We thus examined whether BMAL1-KD influenced CRC cell proliferation by using an MTT Cell Proliferation and Viability Assay (Figure 3A) and cytometry cell counts (Figure 3B).

In primary CRC cells, corresponding to their higher AKT/mTOR activity, MTT analysis showed that HCT116 BMAL1-KD cells exhibited greater proliferation from 72h in culture, compared to its control (72h, p=0.0078; 96h, p<0.0001). This result was confirmed by cell counts, which showed more HCT116 BMAL1-KD cells after 48h compared to control (48h, p=0.0090; 72h, p=0.0317; 96h, p<0.0001). In contrast, despite increased AKT/mTOR activity, the SW480 BMAL1-KD cell line did not show greater cell proliferation compared to its control. At 96h, cytometry cell counts even revealed fewer BMAL1-KD cells compared to control (p=0.0259).

In the metastatic SW620 BMAL1-KD cell line, there was slight increase of AKT activity but without evident increase of mTOR activity. However, this cell line did proliferate faster at 96h when compared to control (MTT: p=0.0460; cell count: p=0.0090).

### BMAL1-KD increased senescence only in SW480 cells

Increased mTOR activity also leads to accelerated aging under certain conditions, as shown in *BMAL1* knockout mice [25–27]. SW480 BMAL1-KD cells demonstrated increased mTOR activity but no increased cellular proliferation, which led us to check whether BMAL1-KD induced senescence in these SW480 cells.

**Figure 4:**
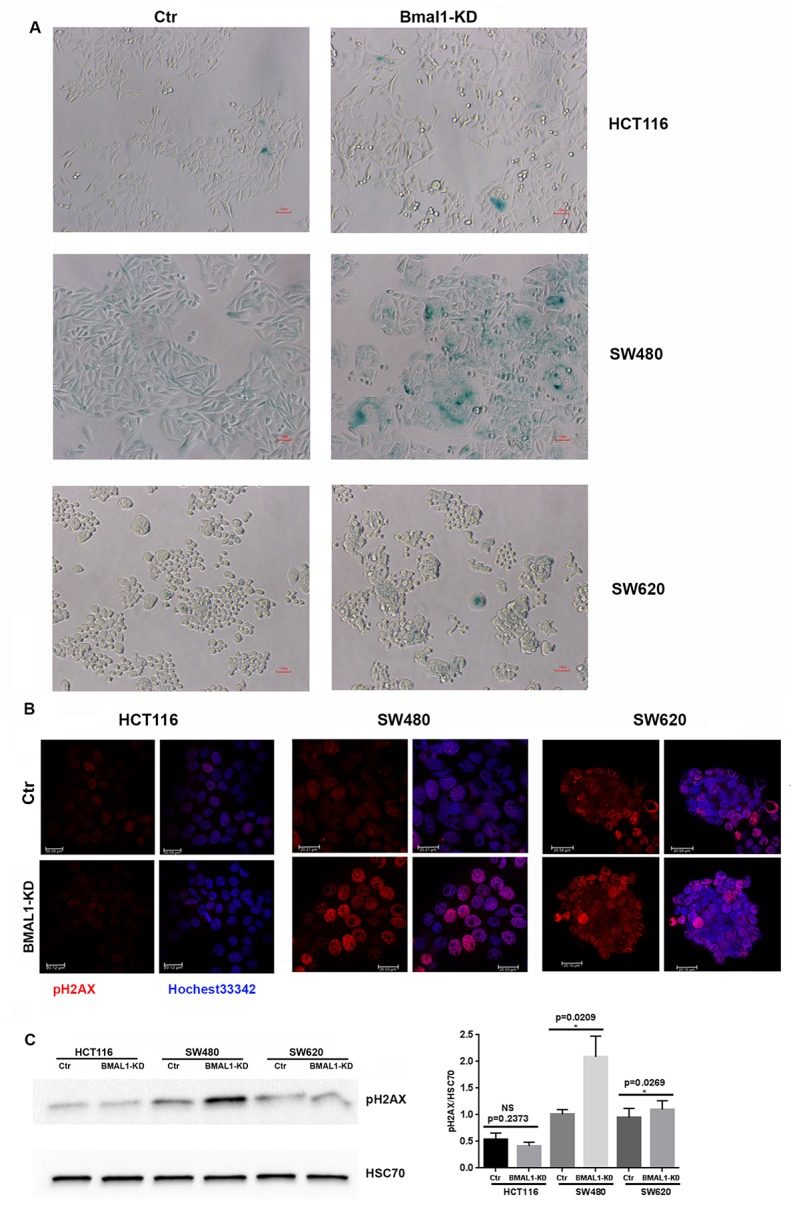
**BMAL1-KD increased senescence in SW480 BMAL1-KD but not in HCT116 BMAL1-KD and SW620 BMAL1-KD cells.** (**A**) Senescence-associated β-galactosidase (SA-β-gal) activity was obviously increased in SW480 BMAL1-KD cells, but not in HCT116 BMAL1-KD nor in SW620 BMAL1-KD cells. SA-β-gal activity was measured by β-galactosidase staining (blue). Scale Bar represents 10 μM. Representative staining of three independent experiments was shown. (**B**) Immunofluorescence identified phosphorylated H2AX (pH2AX, red) in cell nuclei (Hoechst 33342, blue) of BMAL1-KD and control CRC cell lines. Representative staining of three independent experiments were shown. Scale bar, 20 μm. (**C**) Western-blot revealed significant increase of pH2AX mainly in SW480 BMAL1-KD cells. *Left*, a representative immunoblot of three independent experiments was shown. *Right*, Bar charts represented pH2AX expression level normalized to HSC70 (n=7; *p<0.05; ***p<0.001). All data are shown as means ± SEM.

Of the three BMAL1-KD CRC cell lines, only SW480 BMAL1-KD cells showed an obvious increase of cell senescence, as identified by senescence-associated β-galactosidase activity (SA-β-gal) staining. These SA-β-gal-positive cells also demonstrated other senescence related alterations: enlarged cell size and flattened shape (Figure 4A). In contrast, compared to their controls, there was no evident increase in cellular senescence in HCT116 BMAL1-KD and SW620 BMAL1-KD lines.

Another common indicator of senescent cells is the marker of DNA double-strand breaks (DSB), phosphorylated H2AX (pH2AX) [29, 30]. Immunofluorescence (Figure 4B) and western blot (Figure 4C) analyses showed that phosphorylated H2AX was increased strongly in SW480 BMAL1-KD cells (p=0.0209) and only slightly in the SW620 BMAL1-KD cell line (p=0.0269).

**Figure 5:**
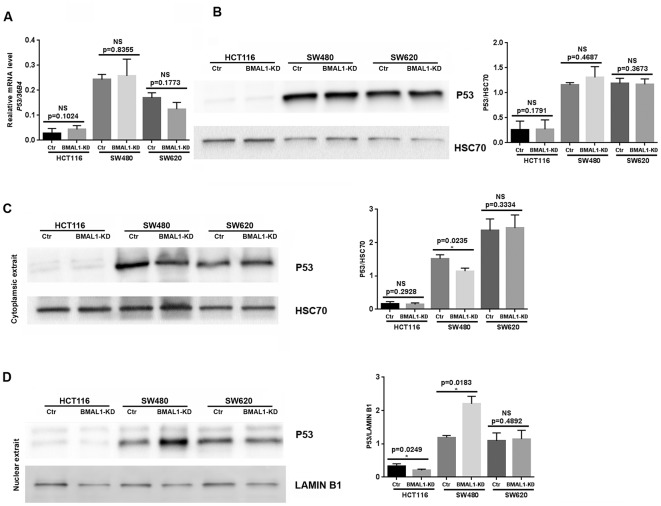
**P53 expression status in CRC BMAL1-KD cell lines.** (**A**) Quantitative RT-PCR revealed that no significant change of P53 mRNA levels in the three CRC cell lines. *36B4* was used as a quantitative reference for all quantitative RT-PCR analyses (n=8). (**B**) Western-blot analysis revealed no significant change of P53 protein levels in the three CRC cell lines (n=7). (C and D) Cytoplasmic (**C**) and nuclear (**D**) extracts from BMAL1-KD and control cell lines were analyzed by western-blot. Only SW480 BMAL1-KD cells exhibited a significant decrease of cytoplasmic P53 (n=5; *p<0.05) associated with a significant increased nuclear P53 (n=5; *p<0.05). *Left*, a representative immunoblot of 5 independent experiments was shown. *Right*, Bar charts represented P53 expression level normalized to HSC70 or LAMIN B1. All data are shown as means ± SEM.

Cell senescence is also mediated by, and can induce, P53/P21 activation [31]. Although P53 mRNA (Figure 5A, qRT-PCR) and protein (Figure 5B, western blot) levels did not change in the three BMAL1-KD CRC cell lines, there was a change of P53 localization in SW480 BMAL1-KD cells. Compared to control, cytoplasmic P53 decreased (p=0.0235; Figure 5C) and P53 nuclear expression increased (p=0.0183; Figure 5D) in SW480 BMAL1-KD cells, suggesting P53 activation. In contrast, P53 cytoplasmic expression did not change in HCT116 BMAL1-KD and SW620 BMAL1-KD cells. Moreover, P53 nuclear localization in HCT116 BMAL1-KD cell line even decreased compared to its own control (p=0.0249; Figure 5D).

**Figure 6:**
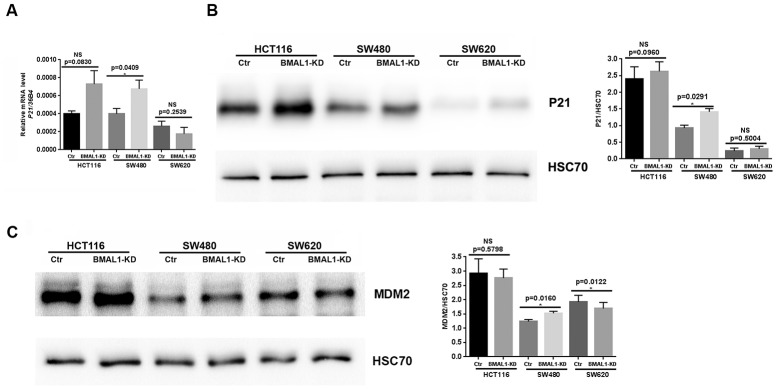
**P21 and MDM2 expression status in CRC BMAL1-KD cell lines.** (**A**) Quantitative RT-PCR revealed a significant increase of P21 mRNA in SW480 BMAL1-KD cells (n=6; *p<0.05) but not in HCT116 BMAL1-KD and SW620 BMAL1-KD cells. (**B**) Western-blot analysis revealed a significant increase of P21 protein in SW480 BMAL1-KD cells (n=4; *p<0.05) but not in HCT116 BMAL1-KD and SW620 BMAL1-KD cells. (**C**) Western-blot analysis revealed increased MDM2 protein in SW480 BMAL1-KD cells (n=5; *p<0.05) but not in HCT116 BMAL1-KD and SW620 BMAL1-KD cells. *Left*, a representative immunoblot of different independent experiments is shown. *Right*, Bar charts represent P21 or MDM2 expression normalized to HSC70. All data are shown as mean ± SEM.

To confirm the link between cellular senescence and P53 expression in our cell lines, we analyzed expression of P53 targets, P21 and MDM2 (murine double minute homolog 2). In agreement with the different P53 activation, qRT-PCR (Figure 6A, p=0.0409) and western-blot results revealed increased P21 (Figure 6B, p=0.0291) and MDM2 (p=0.0160; Figure 6C) expression only in SW480 BMAL1-KD cells compared to control. Moreover, MDM2 expression was even decreased in SW620 BMAL1-KD cells (p=0.0122).

In summary, after BMAL1-KD cellular senescence was only induced in one primary CRC line (SW480) in association with nuclear translocation of P53 and increased expression of P21 and MDM2.

### BMAL1-KD increased apoptosis and P53 activation only in HCT116 cells

The previous results were performed on BMAL1-KD cell lines which were obtained after puromycin selection. However, phenomena such as apoptosis, will appear transiently after shBMAL1 begins to be expressed. GFP gene is incorporated in the lentivirus construction as a reporter of shRNA expression, and it becomes visible 24h post-transfection, indicating the onset of sh*BMAL1* expression. We thus tested for the onset of apoptosis with Annexin V labeling 48h after lentivirus transduction, i.e. around 24h after shBMAL1 expression begins.

**Figure 7:**
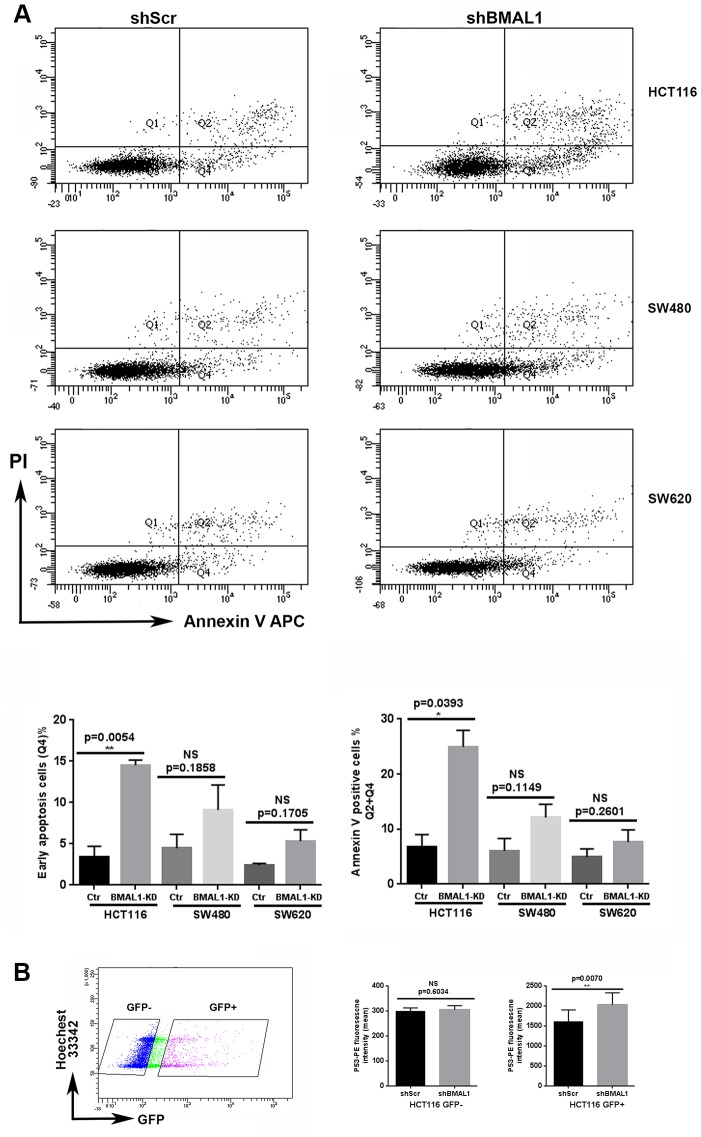
**HCT116-shBMAL1 cells temporarily increased apoptosis and P53 activation after lentivirus transduction.** (**A**) Flow cytometry analysis with Annexin V-APC and propidium iodide staining were applied to determine apoptosis ratio in different shRNA (shBMAL1 or shScr) transduced cells 48h after lentivirus transduction. *Upper panels*, a representative distribution of three independent experiments is shown. *Lower panels*, Graphs represent the percentage of early apoptosis cells (Q4) and total apoptosis cells (Q2+Q4) (3 independent for each analysis). A significant increase of apoptosis ratio is detected only in HCT116 cells after shBMAL1 transduction (**p<0.01). (**B**) Flow cytometry analysis with P53-PE and Hoechst 33342 staining revealed that the nuclei of HCT116 shBMA1 transduced cells (GFP positive population) exhibited an increased P53 expression compared to the nuclei of HCT116 shScr transduced cells (n=3; **p<0.01). *Right*, Graphs represent the mean of P53 nuclei expression from three independent experiments. All data are shown as means ± SEM.

Without puromycin selection, only GFP positive (GFP+) cells were considered as shRNA transduced. In the GFP+ population, the cells undergoing early apoptosis, i.e., Annexin V-positive and PI-negative cells, as well as the total apoptosis cells (Annexin V-positive cells) are all increased in shBMAL1 vs. shScr transduced HCT116 cells (p=0.0054 and p= 0.0393). However, shBMAL1 transduced SW480 (p=0.1858and p=0.1149) or SW620 (p=0.1705 and p=0.2601) cells only presented a trend to increase apoptosis (Figure 7A).

For HCT116 cells, we also measured P53 nuclear expression by flow cytometry, 48h after lentivirus transduction. For the GFP+ population, nuclei of shBMAL1 transduced cells exhibited increased P53 expression compared to shScr control cells (p=0.0070). However, this difference was not observed in the GFP negative (shRNA non-transduced) population (Figure 7B).

## DISCUSSION

In this study, we analyzed the consequences of circadian clock perturbation, specifically BMAL1-KD, on human colorectal cancer cell behavior. We used 3 experimental cell models corresponding to different colorectal cancer progression: two primary colorectal carcinoma cell lines (HCT116 and SW480) and SW620, a metastatic colorectal carcinoma cell line derived from the same patient as SW480. Our results reveal that BMAL1-KD triggers distinct cell fates in different colon cancer cell lines rather than the same phenomenon throughout.

### BMAL1-KD appropriately alters expression of other core circadian genes

BMAL1–KD induced similar, and expected, gene expression changes in the two primary CRC cell lines: decreased *NR1D1* and increased *CLOCK* expression, but without modification of *PER2, CRY1* or *CRY2*. Reduced *NR1D1* expression is consistent with the exclusive control of its promoter by CLOCK–BMAL1 binding to E-box regulatory elements [32] and the presence of two BMAL1 binding sites in the gene (Supplementary Figure 1) [33]. This contrasts with only one binding site for BMAL1 in *PER2*, *CRY1* or *CRY2* genes [33], and explains why *NR1D1* expression is more sensitive to the diminution of BMAL1. Similarly, NR1D1 (REV-ERBα) rapidly represses transcription of *BMAL1* and *CLOCK* genes via REV-ERBΑ response elements (RREs) [34, 35]. So, decreased *NR1D1* and increased *CLOCK* expression in the two primary BMAL1 knockdown CRC cells represent correct feedback regulation of core-clock gene expression [20–22].

### BMAL1-KD increases mTOR activity in CRC cells

In the two primary CRC cell lines, BMAL1-KD increased activity of mTOR, a central regulator of cellular metabolism that links cellular energy and nutrients to cell division, growth, quiescence, senescence and death; and which is critically involved in cellular live, for example aging, diabetes and cancer [25, 26, 36]. Our result is coherent with observations in BMAL1 KO mice and the circadian rhythmicity of mTOR signaling [27, 37–39]. Moreover, we also demonstrate that BMAL1-KD increased phosphorylation of the mTOR effector, ribosomal S6 (Figure 2), whose protein kinase, S6K1, rhythmically phosphorylates BMAL1 so that it associates with cellular translational machinery for protein synthesis [39]. Thus our data support the links between BMAL1 and mTOR pathway, which suggests BMAL1 regulation of protein synthesis and the role of circadian timing in cancer development.

### Different CRC cell fates triggered by BMAL1-KD depend not only on increased mTOR activity but also on P53 status of each cell line

*BMAL1* knockout mice show increased mTOR activity, associated with age-related pathology and reduced lifespan, i.e. premature ageing [27, 40, 41]. Therefore, it is not surprising that cell senescence increased after BMAL1 knockdown and increased mTOR activity. However, why did this occur only in SW480 BMAL1-KD, but not HCT116 BMAL1-KD cells?

A possible explanation for this variance in cellular senescence between SW480 and HCT116 cells is their different P53 status. The HCT116 cell line expresses wild type (WT) P53, whereas the SW480 cell line carries a mutant P53 (R273H and P309S; mP53) which is only partially functional, such as inducing P21 expression [42]. Knockdown of this mP53 decreases colorectal cancer malignancy, indicating its important role in CRC development [43]. Blagosklonny et al. hypothesized that maximal P53 activity blocks mTOR, causing cellular quiescence or death, whereas partial P53 activity sustains mTOR activity and causes senescence [23–26]. In keeping with this hypothesis, we propose that the different P53 status (WT vs mutant) of HCT116 and SW480 cells underlies the different cell fates induced by mTOR activity after BMAL1-KD.

This proposal is supported by our findings. The reduction of BMAL1 in lentiviral transfected cells depends on the number of shRNA copies that are integrated, and will vary for each cell [44]. Thus just after lentiviral transfection, HCT116 BMAL1-KD and SW480 BMAL1-KD cultures will contain many cell populations each with distinct BMAL1, mTOR and P53 activation according to the number of integrated sh*BMAL1* copies. Thus, in HCT116 cells which integrate a high number of sh*BMAL1* copies, there will be high WT P53 activation, which can induce rapid apoptosis, as indeed we observed 24h after sh*BMAL1* expression (Figure 7). Consequently, during the one-week puromycin selection for stable BMAL1-KD, the HCT116 BMAL1-KD cells with strong P53 activation would be removed by apoptosis, leaving those with less P53. Such P53 expression will in turn induce expression of MDM2, which directs P53 for proteasome degradation to limit its expression level and activity [45]. As our results showed, stable HCT116 BMAL1-KD cells had high MDM2 and low P53 expression, consistent with functional MDM2-P53 negative feedback. Finally, in the absence of increased P53 activity, increased AKT and mTOR activity in the stable HCT116 BMAL1-KD cells can increase cellular proliferation (Figure 3).

Alternatively, for SW480 cells, those which integrate many sh*BMAL1* copies will stimulate mP53 activity, as demonstrated by nuclear translocation and expression of P21 and MDM2 (Figure 6). But mP53 has only partial function [42], as confirmed in our results by low expression of MDM2, which would limit MDM2-P53 negative feedback and explain the higher P53 expression in SW480 cells than HCT116 cells. Consequently, mTOR activity is sustained and provokes cell senescence. SW480 BMAL1-KD cells with fewer integrated sh*BMAL1* will only moderately activate mP53, not block mTOR and promote cell survival or proliferation. Our results, showing only a relatively small decrease in SW480 BMAL1-KD cell counts (Figure 3) despite profound cellular senescence (Figure 4), are consistent with cultures being transduced by a range of sh*BMAL1* copies.

### Effect/influence of BMAL1 KD on CRC cell life requires functional circadian rhythm

In contrast to the two primary CRC cell lines, metastatic SW620 cells showed very little response to BMAL1-KD: a small increase in phosphorylated AKT without associated mTOR or S6 activation (Figures 2). A likely explanation for this muted response is an abnormal circadian timing system and low baseline expression of BMAL1 and other cell regulatory proteins (e.g. AKT, P21) in this cell line. In contrast to the two primary CRC cell lines (HCT116 and SW480), the metastatic cell line SW620 has severely diminished core-clock gene oscillation, indicating dysfunction of the transcription-translation feedback loops among different circadian proteins [20–22]. Although SW480 and SW620 cell lines were derived from primary and metastatic sites of the same patient, there was only 5.5% overlap of genes with oscillating expression profiles, indicating the loss of intact circadian clock during tumor progression [22]. Consistent with this, BMAL1 knockdown in metastatic SW620 cells did not alter *NR1D1* and *CLOCK* expression, in contrast to the two primary CRC cells. Thus, a dysregulated core clock in SW620 cells, as indicated by poor oscillation of clock-controlled genes and low level of BMAL1 protein can explain why BMAL1-KD had less influence on SW620 cells than on SW480 cells in our experiments.

### BMAL1 KD-associated oxidative stress reveals impaired P53 signaling in SW620 cells

Global deletion of BMAL1 induces an aging phenotype associated with oxidative stress [46], which damages DNA, including DNA strand breaks.

In stable HCT116 BMAL1-KD cells there was no evident oxidative stress (H2AX phosphorylation), consistent with vulnerable HCT116 BMAL1-KD cells with high WT P53 activation being eliminated by apoptosis just after sh*BMAL1* transduction, and P53 activation in surviving cells being blocked by MDM2-P53 negative feedback.

**Figure 8:**
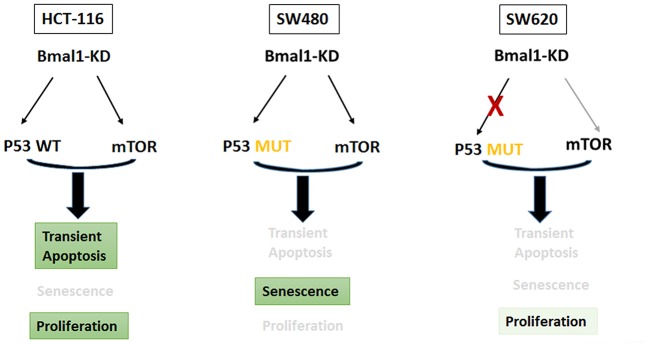
**BMAL1-KD modified the delicate equilibrium between AKT/mTOR and P21/P53 pathways, which triggered the different cell fates based on distinct P53 and circadian rhythm status of every CRC cell line.**

In contrast, our results show phosphorylated H2AX in SW480 and SW620 BMAL1–KD cells, indicating oxidative stress. In SW480 BMAL1-KO cells, there was a large increase in phosphorylated H2AX and mP53 activation. In contrast, SW620 BMAL1-KD cells only slightly increased phosphorylated H2AX but without associated mP53 activation: no P53 nuclear translocation or P21 expression, and even decreased MDM2 expression (Figures 4–6), which suggested impaired P53 signaling in SW620 cells. In the absence of appropriate P53 activation and its induction of apoptosis or senescence, the moderate increase of AKT activity permits faster growth (Figure 8).

## CONCLUSIONS

Briefly, our work provides a potential explanation for the different cell fates induced by BMAL1 knockdown in CRC cells, which is based on increased mTOR activation and different P53 status. However, these CRC cells also contain mutations in other cancer related genes (HCT116: *KRAS, PIK3CA, CTNNB1, BRCA2, CDKN2A* etc.; SW480 or SW620: *KRAS and APC* etc.) [47], which constitute a unique pathological context in every CRC cell. Thus, in addition to differences in core circadian clock status, P53 regulation and basal kinase activity that we demonstrate, all these distinct mutations (including in *P53*) will contribute to the different cell fate induced by BMAL1 knockdown. Altogether, this work reveals the important role of BMAL1 in CRC cell behavior, in particular primary CRC cell fate decision. Knockdown of BMAL1 expression at different levels could potentially commit primary colon cancer cells towards different cell fates.

## MATERIALS AND METHODS

### Cell culture

HCT116, SW480 and SW620 cells were cultured in Dulbecco’s modified Eagle medium (DMEM) supplemented with GlutaMAX (GIBCO, Life Technology, CA, USA) and 10% fetal bovine serum (FBS, Hyclone, UT, USA).

### The shRNA sequence cloning in a lentiviral vector

A short hairpin RNA sequence (shRNA) against human BMAL1 (shBMAL1; Forward: 5'-CCGGAGAACCCAGGTTATCCATATTCTGCAGAATATGGATAACCTGGGTTCTTTTTT-3'; Reverse: 5'-CTAGAAAAAAAGAACCCAGGTTATCCATATTC TGCAGAATATGGATAACCTGGGTTCT-3') or a control scrambled sequence (shScr) were inserted separately into a lentiviral vector (pLKO-shBMAL1-GFP-puro or pLKO-Scr-GFP-puro), which also encode the reporter protein GFP and the puromycin resistance gene.

### Lentivirus production and cell transduction

Lentivirus particles were produced as previously described [48] but without concentration. After lentiviral production, the lentivirus supernatant was filtered with 0.45 μm filters and stored at -80°C. For target cell transduction, 1 mL filtered viral supernatant mixed with 1 mL DMEM (10% Fetal Bovine Serum, FBS) was added to each well of 6-well plates containing 50% confluent cells in the presence of 8 μg/mL polybrene (Sigma, MO, USA) and incubated with for 24 hours.

Transduced cells were either used directly 48h after transfection, or after selection by adding 2 μg/mL puromycin (Thermo Fisher) to the medium 72h after transfection and culturing for 1 week. After puromycin selection, flow cytometry analysis was performed to confirm that the entire cell population was GFP positive. These GFP positive cells, named as BMAL1-KD or control (Ctr) cells, were used for subsequent analysis.

Analyses with or without puromycin selection are repeated with the cells from three independent lentivirus transductions.

### MTT cell proliferation assay

The BMAL1-KD or control CRC cell lines were seeded in 96-well plates at an initial density of 2x10^3^ cells per well. Cell proliferation was measured daily during 4 days by Vybrant MTT Cell Proliferation Assay Kit (V13154, Molecular Probes, Invitrogen). Every time point was repeated 3-4 times in independent experiments. Two-way ANOVA was used for statistical analysis of 8 independent experiments.

### Cell proliferation curve analysis

The BMAL1-KD or control CRC cell lines were seeded in 24-well plates at an initial density of 1x10^4^ cells per well. After trypsinization (Trypsin-EDTA, Thermo Fisher, MA, USA) and suspension in PBS containing 0.5% bovine serum albumin (BSA) and 2 mM EDTA, the number of living cells from each well was counted over 4 days by a Miltenyi Biotec AutoMACS cytometry. Three independent experiments were statistically analyzed by two-way ANOVA.

### Quantitative RT-PCR (qRT-PCR)

Cells were collected and total RNA was extracted as previously described [49]. Reverse transcription was performed with Superscript II RT-kit (Invitrogen, CA, USA). Quantitative real time PCR was performed by using LightCycler 480 SYBR Green I master kit (Roche, Bâle, Switzerland). Primers of *BMAL1* and *36B4* were used for gene amplification were previously described [49]. *P21* primer: Forward-GACACCACTGGAGGGTGACT and Reverse-CAGGTCCACATGGTCTTCCT. *P53* primer: Forward-GTTCCGAGAGCTGAATGAGG and Reverse-TCTGAGTCAGGCCCTTCTGT.

The relative quantification of target RNA by using *36B4* as a reference was computed with the Relquant software (Roche, Bâle, Switzerland) with the “delta delta Ct” (ΔΔCt) method. T-test was used for statistical analysis.

### Flow cytometry analysis of S6 phosphorylation

Cells were detached with Trypsin-EDTA (Thermo Fisher, MA, USA), fixed in ice-cold 70% ethanol, washed twice in ice-cold PBS and then centrifuged at 300g for 10 min. Cells were incubated with anti-Phospho-S6-APC (#14733; Cell Signaling, MA, USA) in PBS containing 0.5% bovine serum albumin (BSA) and 2 mM EDTA for 30 min at 4°C and labelled with Alexa Fluor® 647 or Alexa Fluor® 546 conjugated secondary antibodies (Molecular Probes) and Hoechst 33342 (B2261, Sigma MO, USA). After labelling, cells were washed once time and analyzed in an LSR Fortessa™ cell analyzer (Becton Dickinson, NJ, USA).

### Flow cytometry analysis of nuclear P53 expression

Cells were trypsinized and centrifuged at 1200 rpm for 5 min and suspended very gently in citrate solution (0.1% Trisodium citrate and 0.058% NaCl, pH=7.6) containing 0.001% NP40 and 10 mg/mL Hoechst 33342, incubated for at least 2h (maximum 24h) at 4°C to extract and stain cell nuclei. Cell nuclei were then incubated with anti-P53-PE (130-109-570, Miltenyi Biotec) in a PBS solution containing 0.5% BSA and 2 mM EDTA for 30 min at 4°C. After washing, nuclear P53 expression was analyzed by flow cytometry in an LSR Fortessa^TM^ cell analyzer (Becton Dickinson, NJ, USA). T-test was used for statistical analysis.

### Apoptosis assay

48h after viral transduction, transfected cells were trypsinized and then 10^6^ cells were washed in 1 mL of 1x Binding Buffer (130-092-820, Miltenyi Biotec, Germany) and centrifuged at 300g for 10 min. After 15 min incubation with anti-Annexin V-Alexa Fluor® 647 (640943, BioLegend, CA, USA), cells were washed and suspended in 500 μL 1x binding buffer, 10 mg/mL propidium iodide (PI) solution was added immediately prior to analysis by flow cytometry. All the experiments or solution are realized at 4°C. T-test was used for statistical analysis.

### Immunofluorescence and confocal microscopy

The cells were cultured on 17 mm coverslip until desired confluence (around 50%), fixed with 4% PFA, permeabilized with 0.1% Triton and then blocked with 0.3% BSA before being incubated with primary (Anti-pH2AX 9718, Cell Signaling) and secondary antibodies. The confocal images were captured by a confocal LEICA SP5-AOBS microscope with a 63X/1.4 NA oil-immersion objective. Hoechst 33342 was used for nuclear staining.

### Cytoplasmic and nuclear extracts preparation

Cytoplasmic and nuclear extracts of different cell lines were separated and prepared with NE-PER™ Nuclear and Cytoplasmic Extraction Reagents (78833, Thermo Fisher) by following the kit instruction. The different extracts were stored at -80°C until western-blot analysis by anti-P53 (2527, Cell signaling). HSC70 expression was used as a control of cytoplasmic protein loading (SPA-815, Stressgen) and LAMIN B1 (ab16048, Abcam) was used as nuclear protein loading.

### Western blot

Western blot analysis was performed with sodium dodecyl sulfate polyacrylamide gel electrophoresis (SDS-PAGE) as previously described [49] with different primary antibodies: Anti-HSC70 (SPA-815, Stressgen), or anti-BMAL1 (14020), anti-Phospho-S6 ribosomal protein (Ser240/244; 5456), anti-S6 ribosomal protein (2317), anti-phospho-AKT (Ser473; 4060), anti-AKT(4691), anti-pmTOR (Ser2448; 2971), anti-mTOR (2972), anti-P21 Waf1/Cip1 (2947), anti-MDM2 (86934), anti-P53 (2527) all from Cell Signaling Technology. The results were quantified by Image J. T-test was used for statistical analysis.

## Supplementary Material

Supplementary Figure 1
