# Supplementary material for: BMAL1 knockdown triggers different colon carcinoma cell fates by altering the delicate equilibrium between AKT/mTOR and P53/P21 pathways
Source: Aging (Albany NY). 2020 May 10;12(9):8067–83. doi: 10.18632/aging.103124 (PMC7244025; doi:10.18632/aging.103124)
Supplement: Supplementary Figure 1 [file aging-12-103124-s001..pdf]

SUPPLEMENTARY FIGURE

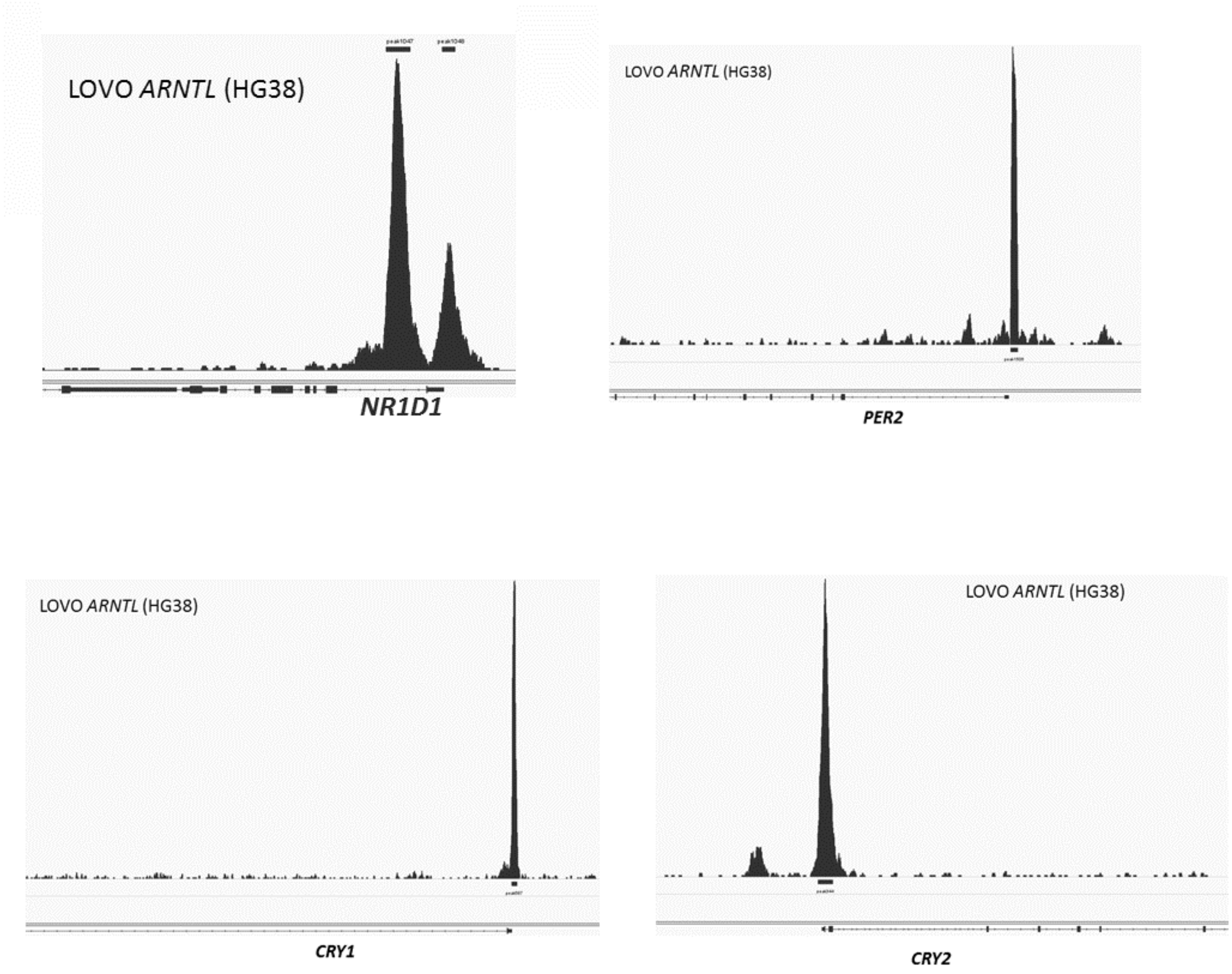

Supplementary Figure 1. Two binding sites for BMAL1 in *NR1D1*gene have been mapped in LOVO CRC cell by in silicon CHIP-seq analysis, but only one binding site of *PER* or *CRY* was identified.
